# A Federated Derivative Cubature Kalman Filter for IMU-UWB Indoor Positioning

**DOI:** 10.3390/s20123514

**Published:** 2020-06-21

**Authors:** Chengyang He, Chao Tang, Chengpu Yu

**Affiliations:** 1 School of Automation, Beijing Institute of Technology, Beijing 100081, China; 3120170434@bit.edu.cn (C.T.); yuchengpu@bit.edu.cn (C.Y.); 2 Beijing Institute of Technology Chongqing Innovation Center, Chongqing 401135, China

**Keywords:** federated derivative cubature Kalman filtering, information fusion, indoor positioning

## Abstract

The inertial measurement unit and ultra-wide band signal (IMU-UWB) combined indoor positioning system has a nonlinear state equation and a linear measurement equation. In order to improve the computational efficiency and the localization performance in terms of the estimation accuracy, the federated derivative cubature Kalman filtering (FDCKF) method is proposed by combining the traditional Kalman filtering and the cubature Kalman filtering. By implementing the proposed FDCKF method, the observations of the UWB and the IMU can be effectively fused; particularly, the IMU can be continuously calibrated by UWB so that it does not generate cumulative errors. Finally, the effectiveness of the proposed algorithm is demonstrated through numerical simulations, in which FDCKF was compared with the federated cubature Kalman filter (FCKF) and the federated unscented Kalman filter (FUKF), respectively.

## 1. Introduction

With the development of science and society, more and more intelligent products have entered in people’s lives, and the most representative of them are intelligent robots, such as cleaning robots, storage robots, and service robots. Most intelligent robots are mainly used in indoor scenarios such as home services, factory logistics, and business services. Therefore, it is of great significance to study high-precision indoor positioning techniques.

The current indoor positioning solutions include WiFi [1], Bluetooth [2], ultra-wide band (UWB) [3,4,5], ZigBee [6], inertial measurement unit (IMU) [7,8,9], and so on. The inertial navigation system is a self-contained system that calculates the position, velocity, and attitude of a vehicle from the outputs of inertial measurement units (IMUs). However, IMUs have drift errors, and their positioning errors increase unrestrictedly with time [10]. Therefore, the IMU as a positioning sensor alone is only effective for a short time. If it needs to work for a long time, other sensors need to be introduced to continuously correct the accumulated error of the IMU. Compared with other indoor positioning solutions, UWB signals have the characteristics of strong anti-multipath effect, low energy consumption, low power consumption, and comparatively high positioning accuracy. Therefore, it is a popular indoor positioning solution with high comprehensive performance. Thanks to the miniature of single chip transceiver [11], UWB positioning system has been adopted to provide accurate location information for multi-agent systems [12,13]. The disadvantage of UWB is that this solution cannot obtain accurate bearing information in an indoor environment. Due to the fact that IMU information and UWB information are complementary [14,15], the fusion of IMU and UWB information can overcome the shortcomings of two independent systems, such as the lack of bearing information of UWB and the long-term drift error of IMU. It also enables to sufficiently exploit the advantages of both stand alone systems, such as UWB’s positioning accuracy does not change over time, and IMU’s short time positioning accuracy is extremely high [16]. Fusion of multiple sensors information and complete real-time fault diagnosis is currently the most popular solution [17].

There are many algorithms for information fusion in the literature such as the filtering based method [18,19,20], the statistical inference method [21,22], and the artificial intelligence method [23]. Among them, Extended Kalman Filter (EKF) is the most commonly used state estimation algorithm for nonlinear systems [24]. This algorithm uses an approximate idea to transform a nonlinear system into a linear system through Taylor expansion [25]. However, when the system’s non-linearity is high, EKF will produce a large truncation error, resulting in inaccurate fusion results. The Unscented Kalman Filter (UKF) uses a finite number of sigma points to propagate the probability density function of state distribution through the nonlinear dynamics of a system [26]. Therefore, UKF is one of the most commonly used methods for attitude estimation [27] and multi-sensor information fusion [28]. In high-dimensional systems, UKF needs to adjust parameters reasonably to obtain better filtering effects, which turns out to be difficult in practice. Ienkaran Arasaratnam and Simon Haykin recently proposed the cubature Kalman filter (CKF) [29] which has been the closest approximation until now to Bayesian filtering in theory. A prominent advantage of CKF is that it is mathematically rigorous, which is reflected in the fact that CKF is based on the third-degree spherical-radial cubature rule. A unique characteristic of the CKF is that the spherical radial cubature rule leads to an even number of equally weighted cubature points. These cubature points are distributed on a sphere centered on the real point [30,31]. The state estimation and target tracking based on cubature Kalman have gradually received more and more attention from scholars [32]. Large-scale system will lead to the problem of high calculation complexity and large calculation burden [33]. It is an efficient solution to decompose the large system into multiple subsystems for distributed and decentralized calculation [34,35]. In order to improve system performance, it is necessary to adopt a framework of decentralized Kalman filtering [36]. The federated filter uses a two-stage filtering structure, which reduces the amount of calculation and improves the system’s fault tolerance [37]. To sum up, a distributed framework for federated filtering shall be proposed in this paper, where the derivative CKFs are used as sub-filters to fuse UWB and IMU information.

The rest of the paper is organized as follows. In Section 2, we introduce practical systems with both UWB and IMU sensors [38]. Section 3 proposes an estimation fusion algorithm based on the derivative CKF in the federated filtering framework which is called the federated derivative cubature Kalman filter (FDCKF). The derivative CKF uses KF to simplify the calculation of the measurement update without reducing the estimation accuracy. In addition, inspired by [39,40], this paper also applies Singular Value Decomposition (SVD) decomposition to the DCKF algorithm to improve the convergence of the algorithm and avoid ill-posed problems [41,42] during the Cholesky decomposition. In Section 4, the proposed algorithm is verified through numerical examples on the odometer based motion system. Finally, conclusions are made in Section 5.

The terms of the filtering algorithm appearing in this section will be explained respectively in Table 1.

**Figure 1:**
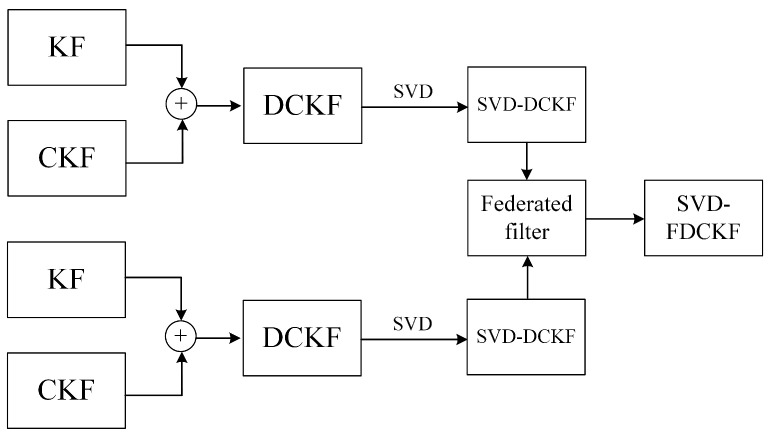
Relationship between filtering algorithms.

The relationship between these filtering algorithms is shown in Figure 1.

This article proposes a new IMU-UWB fusion scheme called Singular Value Decomposition (SVD)-FDCKF, which continuously corrects the IMU through UWB positioning information to avoid the accumulation of IMU drift errors. With the assistance of UWB, the high positioning accuracy of the IMU in a short time can be fully utilized.

## 2. IMU-UWB Mobile Robot System Model

**Figure 2:**
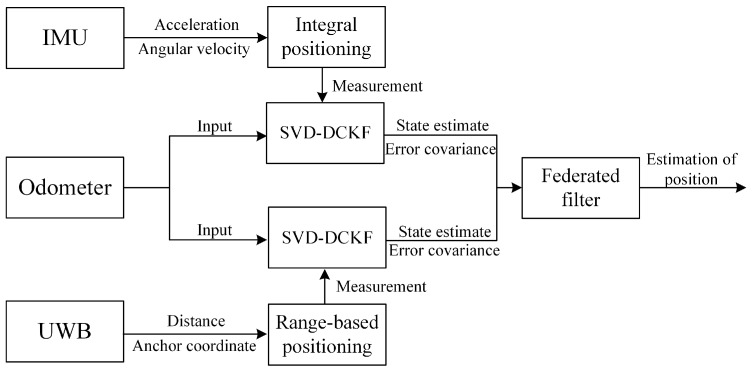
Inertial measurement unit and ultra-wide band signal (IMU-UWB) indoor positioning via the Singular Value Decomposition (SVD)-federated derivative cubature Kalman filter (FDCKF).

As shown in Figure 2, the system used in this paper is a mobile robot system equipped with UWB sensors and IMU sensors. For a mobile robot running in an indoor environment, precise position information is necessary for performing complex mobile tasks. The IMU information can provide accurate position information and direction information of the mobile robot in a short time. Over time, accelerometer errors in the IMU can lead to accumulation of drift errors. Although UWB information cannot provide complete pose information, its positioning results are relatively stable, indicating that the positioning accuracy will not change with time. The mobile robot considered in this article is a wheeled mobile robot, so it is also equipped with an odometer, which can obtain the speed, acceleration, and posture information of the mobile robot by recording the driving mileage of the driving wheels.

**Figure 3:**
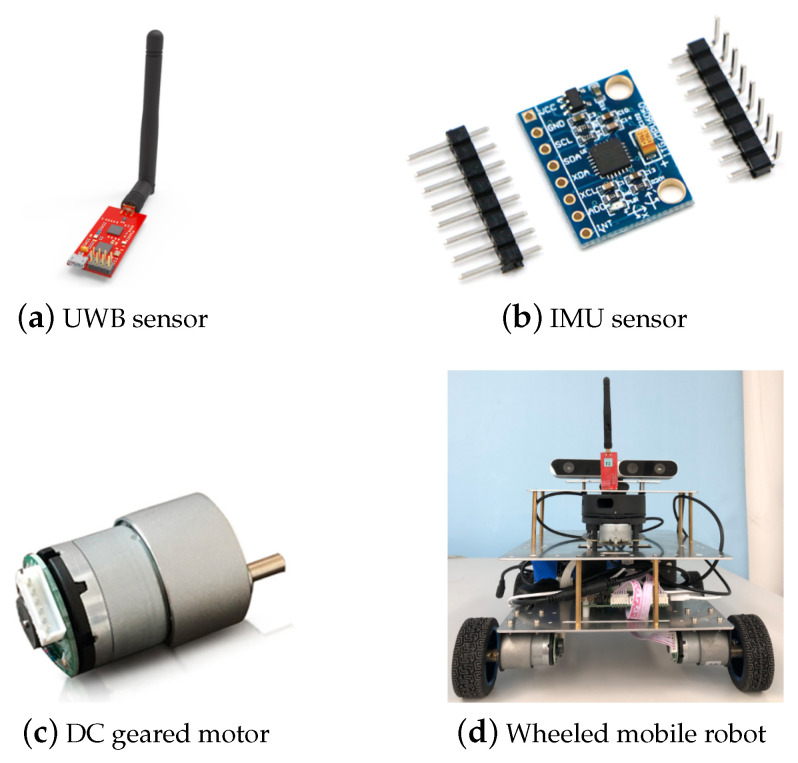
The sensors and mobile robot involved in this article.

The specification of simulated sensors and mobile robot are shown in Figure 3. (a) The UWB sensors are commercial products provided by the company of YCHIOT, and its modole is Mini3s; (b) we chose a very popular spatial motion sensor chip MPU6050 as the IMU sensor; (c) the odometer is constructed by the DC gear motors MG513 with encoder; (d) the wheeled mobile robot platform realizes accurate positioning by carrying the above sensors. The specific applications and mathematical expressions of these sensors will be described in detail below.

### 2.1. Description of System State Equation

The position change of the mobile robot system is based on the odometer, so we choose the odometer model to describe the current system state. Assuming that the indoor environment is an ideal plane environment, the position of the mobile robot can be expressed as (x,y,θ), where *x* and *y* represent the position coordinates of the robot in the Cartesian coordinate system, and θ represents the yaw angle of the robot. Based on this state information, the mobile robot can be navigated. The system state vector $X_{k}$ is defined as follows:

(1) $$\begin{array}{c} X_{k}={\left[x_{k},y_{k},\theta_{k}\right]}^{T} \end{array}$$

where the subscript *k* indicates the value of the state at time *k*. According to the characteristics of the odometer, state-space model for the mobile robot is given as:

(2) $$\begin{array}{c} X_{k+1}=\left[\begin{array}{c} x{}_{k}+M{}_{k}/\gamma {}_{k}\left(cos(\theta {}_{k}+\gamma {}_{k}\right)-cos\theta {}_{k})\\ y{}_{k}+M{}_{k}/\gamma {}_{k}\left(sin(\theta {}_{k}+\gamma {}_{k}\right)-sin\theta {}_{k})\\ \theta {}_{k}+\gamma {}_{k} \end{array}\right]+w_{k} \end{array}$$

where $M_{k}$ is the distance recorded by odometer, $\gamma_{k}$ is the change amount of the yaw angle of the mobile robot, which can be obtained by the odometer based on the difference between the left and right wheel movement distances. The $w_{k}$ is the Gaussian white noise with its variance $Q_{k}$ being expressed by:

(3) $$\begin{array}{c} E\left[w_{k}w_{k}^{T}\right]=Q_{k} \end{array}$$

The variables $M_{k}$ and $\gamma_{k}$ included in the above state equation do not belong to the state variable $X_{k}$ of the system; instead, they are used as inputs $u_{k}$ in this state equation. For wheeled mobile robots, the odometer’s change value of the left wheel $△M_{l,k}$ and right wheel $△M_{r,k}$ can be obtained separately. The input of the state equation can be obtained by the following formula:

(4) $$\begin{array}{c} M_{k}=\left(△M_{r,k}+△M_{l,k}\right)/2\\ \gamma_{k}=\left(△M_{r,k}-△M_{l,k}\right)/L \end{array}$$

where *L* is the distance between the driving wheels of the wheeled mobile robot. Consequently, it can be seen from Equation (2) that the system’s state equation is a nonlinear equation with inputs.

### 2.2. Description of System Measurement Equations

The measurement equations considered in this paper are obtained according to the UWB module and the IMU module, respectively. The UWB module can calculate the position of the mobile robot based on the ranging information, and the IMU module can obtain the position information based on the acceleration integral. Next, it will be described in detail.

#### 2.2.1. IMU Measurement Equation

The IMU consists of a gyroscope and an accelerometer, which are used to measure acceleration and angular velocity, respectively. Integrating the acceleration twice can yield the position information, and similarly integrating the angular velocity can achieve the yaw angle information. However, the measurement results of low-cost commercial accelerometers are of low accuracy, and the acceleration deviation is seriously affected by the working time, i.e., the positioning results will have a large drift error as the working time increases. The data can be collected from the IMU including the position $\left(x_{k},y_{k}\right)$ and the yaw angle $\left(\theta_{k}\right)$ of the mobile robot.

The measurement equation of the system based on the IMU is shown as follows:

(5) $$\begin{array}{cc} \left[\begin{array}{c} Z_{x,k}\\ Z_{y,k}\\ Z_{\theta ,k} \end{array}\right] & =\left[\begin{array}{ccc} 1 & 0 & 0\\ 0 & 1 & 0\\ 0 & 0 & 1 \end{array}\right]\left[\begin{array}{c} x_{k}\\ y_{k}\\ \theta_{k} \end{array}\right]+\left[\begin{array}{c} v_{1_{x},k}\times \frac{T^{2}}{2}\\ v_{1_{y},k}\times \frac{T^{2}}{2}\\ v_{1_{\theta },k} \end{array}\right] \end{array}$$

where the $v_{1_{x},k}$, $v_{1_{y},k}$, $v_{1_{\theta },k}$ are measurement noise with variance $R_{1,k}$, and *T* denotes the signal sampling period.

(6) $$\begin{array}{c} E\left[v_{1_{x},k}v_{1_{x},k}^{T}\right]=R_{1,k} \end{array}$$

#### 2.2.2. UWB Measurement Equation

In the set up of the UWB positioning system, three UWB sensors are deployed as anchors. We establish a Cartesian coordinate system by accurately measuring the distance and angle between these anchors. A UWB sensor is deployed on the mobile robot as a tag, and the time of arrival (TOA) method is used for distance measurement between the tag and the anchors. The coordinates of the tag are calculated using the distance measurement information and the coordinates of the anchors. Typical TOA based algorithms include trilateration, weighted least square, second order cone programming (SOCP), etc. Since the UWB sensor’s refresh frequency can reach 200 Hz, it can be considered that the UWB positioning result can reflect the mobile robot’s position information (x,y) in real time.

The measurement equation of the UWB localization system is shown as follows:

(7) $$\begin{array}{c} \left[\begin{array}{c} Z_{x,k}\\ Z_{y,k} \end{array}\right]=\left[\begin{array}{ccc} 1 & 0 & 0\\ 0 & 1 & 0 \end{array}\right]\left[\begin{array}{c} x{}_{k}\\ y{}_{k}\\ \theta {}_{k} \end{array}\right]+v{}_{2,k} \end{array}$$

where the $\upsilon_{2,k}$ is the measurement noise and its variance $R_{2,k}$ is expressed as:

(8) $$\begin{array}{c} E\left[v_{2,k}v_{2,k}^{T}\right]=R_{2,k} \end{array}$$

## 3. The Federated Derivative Cubature Kalman Filter

### 3.1. Problem Statement

In the previous section, we have shown the state equations of the moving system to be located and the measurement equations of the IMU-UWB. It is not difficult to see that the system’s state equation is nonlinear, while the measurement equation is linear. Due to the linear nature of the measurement equation, if a combination of federated filtering and CKF filtering is used, a volume of redundant calculations will be generated when the cubature points are propagated through the measurement equation. Meanwhile, the Cholesky decomposition used in CKF requires the matrix to be a positive definite matrix, which has strong limitations. The SVD decomposition can decompose any form of matrix, and can be transformed into the form required by CKF. Therefore, the problem of interest is to design a SVD-FDCKF method so as to improve the computational efficiency and the localization performance in terms of estimation accuracy.

The SVD-FDCKF method developed in this section uses derivative CKF and the distributed framework of federated filtering to excute distributed information fusion. The main framework of the SVD-FDCKF algorithm is shown in Algorithm 1. Further detailed steps and related proofs will be explained in the sequel.
**Algorithm 1** A Cycle of SVD-FDCKF Algorithm to Estimate System State.**Input:**$M_{k}$, $\gamma_{k}$, ${\hat{X}}_{k}$, $P_{k}$, $Z_{k+1}^{1}$, $Z_{k+1}^{2}$**Output:**${\hat{X}}_{k+1}$, $P_{k+1}$ CKF-Time
Update1:Initialize state estimate ${\hat{X}}_{k}$ and the error covariance matrix $P_{k}$.2:Calculate the state cubature points $X_{k}^{j}$ by SVD decomposition.3:Propagating the cubature points $X_{k}^{j}$ through the nonlinear state equation.4:Calculate the predicted state ${\hat{X}}_{k+1|k}^{j}$ and the error covariance $P_{k+1|k}$.KF-MeasurementUpdate5:Calculate the predicted measurements of two sub-filters ${\hat{Z}}_{k+1}^{1}\&{\hat{Z}}_{k+1}^{2}$ at the same time according to KF.6:The state estimates ${\hat{X}}_{k+1}^{1},{\hat{X}}_{k+1}^{2}$ and error covariance matrices $P_{k+1}^{1},P_{k+1}^{2}$ of the two sub-filters are updated separately.7:**return** Fuse two local filters to obtain a better estimation by reset federated filter and return ${\hat{X}}_{k+1}$, $P_{k+1}$.

### 3.2. Algorithm Description

The considered nonlinear discrete-time system is represented by:

(9) $$\begin{array}{cc} X_{k+1} & =f\left(X_{k},u_{k}\right)+w_{k}\\ Z_{k}^{1} & =H_{1}X_{k}+v_{1,k}\\ Z_{k}^{2} & =H_{2}X_{k}+v_{2,k} \end{array}$$

where $X_{k}\in R^{n}$ and $Z_{k}^{i}\in R_{i}^{m}\;\left(i=1,2\right)$ denote respectively the state and measurement vector at time *k*. The state $X_{k}={\left[x_{k},y_{k},\theta_{k}\right]}^{T}$, f(·) is the nonlinear *n*-dimensional vector function, and $H_{i}$ is the measurement matrix. $w_{k}$ and $v_{i,k}$ are the uncorrelated zero-mean Gaussian white noise processes with covariances as:

(10) $$\begin{array}{cc} E[w_{k}w_{k}^{T}] & =Q_{k}\\ E[v_{i,k}v_{i,k}^{T}] & =R_{i,k}\;i=1,2 \end{array}$$

The optimal Bayesian filter (BF) is an ideal tool for state estimation. For nonlinear system state models, Bayesian filtering requires an approximate analysis in practice. When the density functions of the state and the measurement variables conforms to the Gaussian distribution, CKF is an approximate non-linear filtering algorithm closest to BF. The procedure of the proposed SVD decomposition based federated cubature Kalman filter (SVD-FDCKF) is elaborated as follows:

- Step 1. Initialize state estimate ${\hat{X}}_{k}$ and the error covariance matrix $P_{k}$.
- Step 2. Since the error covariance matrix $P_{k}$ is a positive definite matrix, the state cubature points are calculated by SVD decomposition, and its weight as follows:
  (11) $$\begin{array}{cc} P_{k} & =U\Sigma U^{T}=\left(U\sqrt{\Sigma }\right){\left(U\sqrt{\Sigma }\right)}^{T}\\ \; & =S_{k}S_{k}^{T}\\ X_{k}^{j} & =S_{k}\xi_{j}+{\hat{X}}_{k}\;j=1,2,\cdots ,m \end{array}$$
  where *m* is the total number of cubature points. According to Spherical–Radial Cubature law, we have m=2n where *n* is the dimension of the state quantity. $\Sigma =diag(\lambda_{1},\cdots ,\lambda_{n})$ is a diagonal matrix composed of eigenvalues. $\xi_{j}$ is the cubature point which is set to:
  (12) $$\xi_{j}=\left\{\begin{array}{c} \sqrt{n}I_{j}\;j=1,\cdots ,n\\ -\sqrt{n}I_{j}\;j=n+1,\cdots ,2n \end{array}\right.$$
  where $I_{j}$ represents the *j*th column of the identity matrix. The cubature points’ weights are set to:
  (13) $$\begin{array}{c} \omega_{j}=\frac{1}{m} \end{array}$$
- Step 3. Evaluate the propagated cubature points. The transformed cubature points yielded through the nonlinear state model are given as follows:
  (14) $$\begin{array}{c} X_{k+1|k}^{j}=f\left(X_{k}^{j},u_{k}\right)\;j=1,2,\cdots ,m \end{array}$$
- Step 4. Calculate the predicted state and the error covariance as follows:
  (15) $$\begin{array}{cc} {\hat{X}}_{k+1|k} & =\sum_{j=1}^{2n}\omega_{j}X_{k+1|k}^{j}\\ P_{k+1|k} & =\sum_{j=1}^{2n}\omega_{j}X_{k+1|k}^{j}{\left(X_{k+1|k}^{j}\right)}^{T}\\ \; & \;\;\;\;-{\hat{X}}_{k+1|k}{\left({\hat{X}}_{k+1|k}\right)}^{T}+Q_{k} \end{array}$$
  After the time update, the measurement update is then performed.
- Step 5. As the system measurement equation is linear, the KF method is used for the measurement update and the Kalman gain calculation. The predicted measurement can be computed as follows:
  (16) $$\begin{array}{cc} {\hat{Z}}_{k+1}^{i} & =H_{i}{\hat{X}}_{k+1|k} \end{array}$$
- Step 6. The state estimate ${\hat{X}}_{k+1}^{i}$ and the corresponding error covariance matrix $P_{k+1}^{i}$ can be updated by:
  (17) $$\begin{array}{cc} {\hat{X}}_{k+1}^{i} & =E(X_{k+1}|Z_{k+1}^{i})\\ \; & ={\hat{X}}_{k+1|k}+K_{k+1}^{i}\left(Z_{k+1}^{i}-{\hat{Z}}_{k+1}^{i}\right) \end{array}$$
  (18) $$\begin{array}{cc} P_{k+1}^{i} & =cov({\tilde{X}}_{k+1}^{i})\\ \; & =P_{k+1|k}-P_{k+1|k}H_{i}^{T}\\ \; & \;{\left(H_{i}P_{k+1|k}H_{i}^{T}+R_{k+1}\right)}^{-1}H_{i}P_{k+1|k} \end{array}$$
  where ${\tilde{X}}_{k+1}^{i}=X_{k+1}-{\hat{X}}_{k+1}^{i}$ is filtering error. The Kalman gain at time *k* can be obtained as follows:
  (19) $$\begin{array}{c} K_{k+1}^{i}=P_{k+1|k}H_{i}^{T}{\left(H_{i}P_{k+1|k}H_{i}^{T}+R_{k+1}\right)}^{-1} \end{array}$$
- Step 7. Adopt the federated filtering framework to fuse the local estimation information of two local filters to obtain a better estimation:
  (20) $$\begin{array}{cc} P_{k+1}^{g} & ={\left[{\left(P_{k+1}^{1}\right)}^{-1}+{\left(P_{k+1}^{2}\right)}^{-1}\right]}^{-1}\\ {\hat{X}}_{k+1}^{g} & =P_{k+1}^{g}\left[{\left(P_{k+1}^{1}\right)}^{-1}{\hat{X}}_{k+1}^{1}+{\left(P_{k+1}^{2}\right)}^{-1}{\hat{X}}_{k+1}^{2}\right] \end{array}$$
  where $P_{k+1}^{g}$ and ${\hat{X}}_{k+1}^{g}$ in the above formula are used respectively to re-initialize $P_{k}$ and $\hat{X_{k}}$ in Step 1, and then repeat Step 1 to Step 7 for the next sample.

In the process of state estimation, the proposed SVD-FDCKF algorithm was updated by adopting a simple and effective KF method to replace the CKF method which uses extra cubature points to approximate the state mean and covariance for the linear measurement equation. Compared with the CKF method, the proposed algorithm reduces the redundant computation load.

In addition to the applicability of the system proposed in this article, the proposed method can be modified to deal with systems where the state equation is a linear equation and the measurement equation is a nonlinear equation.

**Lemma** **1.** *The set of cubature points have the same sample mean and covariance as the distribution of the system $X_{k}$ in (9).*

**Proof.**  Cubature points are of equal weights about ${\hat{X}}_{k}$. Meanwhile, they are symmetrically selected. From the rules of cubature points’ selection which can be obtain in Formula (11) and (12), it is clear that the sample mean is ${\hat{X}}_{k}$.

Suppose that the sample covariance is *P*. We can conclude

(21) $$\begin{array}{cc} P & =\sum_{j=1}^{2n}w_{j}\left\{X_{k}^{j}-{\hat{X}}_{k}\right\}{\left\{X_{k}^{j}-{\hat{X}}_{k}\right\}}^{T}\\ \; & =\sum_{j=1}^{2n}\frac{1}{m}\left\{S_{k}\xi_{j}\right\}{\left\{S_{k}\xi_{j}\right\}}^{T}\\ \; & =\sum_{j=1}^{2n}\frac{1}{2n}\left\{S_{k}\xi_{j}\xi_{j}^{T}S_{k}^{T}\right\}\\ \; & =S_{k}\sum_{j=1}^{2n}\frac{1}{2n}\left\{\xi_{j}\xi_{j}^{T}\right\}S_{k}^{T} \end{array}$$

From the known conditions (12), we can obtain that $\sum_{j=1}^{2n}\frac{1}{2n}\xi_{j}\xi_{j}^{T}$ is a identity matrix and the equation $S_{k}S_{k}^{T}=P_{k}$. Therefore, we can get $P=P_{k}$ from the above formula. □

**Theorem** **1.** *For the system given in Section 2, the proposed DCKF algorithm has the same estimation error covariance as CKF.*

**Proof.**  In order to facilitate the distinction, the symbols used by the CKF algorithm are superscripted with ‘*c*’.

For non-linear state equations, the state prediction ${\hat{X}}_{k+1|k}$ and the error covariance prediction $P_{k+1|k}$ can be obtained after time updates. Factorize the error covariance and calculate the measurement cubature point as follows:

(22) $$\begin{array}{cc} P_{k+1|k} & =S_{k+1|k}^{c}{S_{k+1|k}^{c}}^{T}\\ X_{k+1|k}^{*,j,c} & =S_{k+1|k}^{c}\xi_{j}^{c}+{\hat{X}}_{k+1|k} \end{array}$$

where $\xi_{j}^{c}$ is the measurement cubature point which is expressed as:

(23) $$\xi_{j}^{c}=\left\{\begin{array}{c} \sqrt{n}I_{j}\;j=1,\cdots ,n\\ -\sqrt{n}I_{j}\;j=n+1,\cdots ,2n \end{array}\right.$$

Cubature points are propagated through the linear observation equation:

(24) $$\begin{array}{c} Z_{k+1}^{j,c}=HX_{k+1|k}^{*,j,c} \end{array}$$

From this, the predicted measurement can be obtained by:

(25) $$\begin{array}{cc} {\hat{Z}}_{k+1}^{c} & =\frac{1}{2n}\sum_{i=1}^{2n}Z_{k+1}^{j,c}\\ \; & =\frac{1}{2n}H\sum_{i=1}^{2n}X_{k+1|k}^{*,j,c}\\ \; & =H{\hat{X}}_{k+1|k} \end{array}$$

By the result in Lemma 1, the measurement covariance of CKF can be derived as:

(26) $$\begin{array}{cc} P_{k+1}^{Z,c} & =\frac{1}{2n}\sum_{j=1}^{2n}Z_{k+1}^{j,c}{\left(Z_{k+1}^{j,c}\right)}^{T}-{\hat{Z}}_{k+1}^{c}{\left({\hat{Z}}_{k+1}^{c}\right)}^{T}+R_{k+1}\\ \; & =\frac{1}{2n}\sum_{j=1}^{2n}HX_{k+1|k}^{*,j,c}{\left(X_{k+1|k}^{*,j,c}\right)}^{T}H^{T}\\ \; & \;\;-H{\hat{X}}_{k+1|k}{\left({\hat{X}}_{k+1|k}\right)}^{T}H^{T}+R_{k+1}\\ \; & =H[\sum_{j=1}^{2n}\frac{1}{2n}X_{k+1|k}^{*,j,c}{\left(X_{k+1|k}^{*,j,c}\right)}^{T}\\ \; & \;-{\hat{X}}_{k+1|k}\sum_{j=1}^{2n}{\left(X_{k+1|k}^{*,j,c}\right)}^{T}-\sum_{j=1}^{2n}X_{k+1|k}^{*,j,c}{\left({\hat{X}}_{k+1|k}\right)}^{T}\\ \; & \;+{\hat{X}}_{k+1|k}{\left({\hat{X}}_{k+1|k}\right)}^{T}]H^{T}+R_{k+1}\\ \; & =H[\sum_{j=1}^{2n}\frac{1}{2n}\left(X_{k+1|k}^{*,j,c}-{\hat{X}}_{k+1|k}\right)\\ \; & \;{\left(X_{k+1|k}^{*,j,c}-{\hat{X}}_{k+1|k}\right)}^{T}]H^{T}+R_{k+1}\\ \; & =HP_{k+1|k}H^{T}+R_{k+1} \end{array}$$

Using the same proof method as (26), we can get the cross-correlation matrix between the predicted state and measurement:

(27) $$\begin{array}{cc} P_{k+1}^{XZ,c} & =\frac{1}{2n}\sum_{j=1}^{2n}X_{k+1|k}^{*,j,c}{\left(Z_{k+1}^{j,c}\right)}^{T}-{\hat{X}}_{k+1|k}{\left({\hat{Z}}_{k+1}^{c}\right)}^{T}\\ \; & =\frac{1}{2n}\sum_{j=1}^{2n}X_{k+1|k}^{*,j,c}{\left(X_{k+1|k}^{*,j,c}\right)}^{T}\\ \; & \;\;\;\;-{\hat{X}}_{k+1|k}{\left({\hat{X}}_{k+1|k}\right)}^{T}H^{T}\\ \; & =[\frac{1}{2n}\sum_{j=1}^{2n}X_{k+1|k}^{*,j,c}{\left(X_{k+1|k}^{*,j,c}\right)}^{T}\\ \; & \;\;\;\;-{\hat{X}}_{k+1|k}{\left({\hat{X}}_{k+1|k}\right)}^{T}]H^{T}\\ \; & =P_{k+1|k}H^{T} \end{array}$$

The Kalman gain of CKF can be obtained by the following formula:

(28) $$\begin{array}{cc} K_{k+1}^{c} & =P_{k+1}^{XZ,c}{\left(P_{k+1}^{Z,c}\right)}^{-1}\\ \; & =P_{k+1|k}H^{T}{\left(HP_{k+1|k}H^{T}+R_{k+1}\right)}^{-1} \end{array}$$

From (19) and (28), we can get $K_{k+1}^{ckf}=K_{k+1}^{i}$, i.e., DCKF and CKF can get the same Kalman gain in the system used in this paper. After getting Kalman gain, we can update the state prediction by:

(29) $$\begin{array}{c} {\hat{X}}_{k+1}^{c}={\hat{X}}_{k+1|k}+K_{k+1}^{c}\left(Z_{k+1}-{\hat{Z}}_{k+1}^{c}\right) \end{array}$$

According to formula (16) and (24), we have $Z_{k+1}^{j,c}={\hat{Z}}_{k+1}^{i}$. It can be found that (17) and (29) are equivalent. Therefore, both the CKF algorithm and the DCKF algorithm proposed in this paper have the comparative estimation accuracy. □

**Theorem** **2.** *The SVD-FDCKF fusion algorithm proposed in this paper yields an optimal estimate of the system state in the sense of minimum variance.*

**Proof.**  The centralized fusion estimation is the optimal estimation of the system state in the sense of minimum variance. Therefore, it is sufficient to prove that the algorithm proposed in this paper is equivalent to the centralized fusion algorithm. Consider the time update first. In the time update part, the algorithm is not performed independently, i.e., $P_{k+1|k}=P_{k+1|k}^{g}$ and ${\hat{X}}_{k+1|k}={\hat{X}}_{k+1|k}^{g}$. Therefore, it is only necessary to prove that the measurement update part is equivalent to the centralized fusion.

Define the centralized fusion measurement equation:

(30) $$\begin{array}{c} Z_{k}=HX_{k}+v_{k} \end{array}$$

where $Z_{k}={\left[Z_{k}^{1},Z_{k}^{2}\right]}^{T}$, $H={\left[H_{1}^{T},H_{2}^{T}\right]}^{T}$, $v_{k}={\left[v_{1,k}^{T},v_{2,k}^{T}\right]}^{T}$ and $R_{k}=diag\left(R_{1,k},R_{2,k}\right)$. The measurement update of centralized fusion can be described as:

(31) $$\begin{array}{cc} \; & {\left(P_{k+1}^{global}\right)}^{-1}={\left(P_{k+1|k}^{g}\right)}^{-1}+H^{T}R_{k+1}^{-1}H \end{array}$$

(32) $$\begin{array}{cc} \; & {\left(P_{k+1}^{global}\right)}^{-1}{\hat{X}}_{k+1}^{global}={\left(P_{k+1|k}^{g}\right)}^{-1}{\hat{X}}_{k+1}^{g}+H^{T}R_{k+1}^{-1}Z_{k+1} \end{array}$$

According to (20), we can obtain that:

(33) $$\begin{array}{cc} ( & P_{k+1}^{g}{)}^{-1}={\left(P_{k+1}^{1}\right)}^{-1}+{\left(P_{k+1}^{2}\right)}^{-1}\\ \; & =\sum_{i=1}^{2}\left[{\left(P_{k+1|k}^{i}\right)}^{-1}+H_{i}^{T}R_{k+1}^{i}H_{i}\right]\\ \; & ={\left(P_{k+1|k}^{g}\right)}^{-1}+\left(H_{1}^{T},H_{2}^{T}\right)\times diag\left({\left(R_{k+1}^{1}\right)}^{-1},{\left(R_{k+1}^{2}\right)}^{-1}\right)\\ \; & \;\;\times {\left(H_{1}^{T},H_{2}^{T}\right)}^{T}\\ \; & ={\left(P_{k+1|k}^{g}\right)}^{-1}+H^{T}R_{k+1}^{-1}H\\ \; & ={\left(P_{k+1}^{global}\right)}^{-1} \end{array}$$

In addition, it can be derived that:

(34) $$\begin{array}{cc} (P_{k+1}^{g} & {)}^{-1}{\hat{X}}_{k+1}^{g}={\left(P_{k+1}^{1}\right)}^{-1}{\hat{X}}_{k+1}^{1}+{\left(P_{k+1}^{2}\right)}^{-1}{\hat{X}}_{k+1}^{2}\\ \; & =\sum_{i=1}^{2}\left[{\left(P_{k+1|k}^{i}\right)}^{-1}{\hat{X}}_{k+1}^{i}+H_{i}^{T}{\left(R_{k+1}^{i}\right)}^{-1}H_{i}{\hat{X}}_{k+1}^{i}\right]\\ \; & ={\left(P_{k+1|k}^{g}\right)}^{-1}{\hat{X}}_{k+1}^{g}+H^{T}R_{k+1}^{-1}Z_{k+1}\\ \; & ={\left(P_{k+1}^{global}\right)}^{-1}{\hat{X}}_{k+1}^{global} \end{array}$$

Then it can be seen from Equations (33) and (34) that the algorithm proposed in this paper is equivalent to the centralized fusion algorithm, implying that the proposed method can achieve the optimal estimation of the system state in the sense of minimum variance. □

### 3.3. Computational Burden Analysis

To evaluate the computational burden of the identification algorithm, we will count the number of multiplication and addition operations of the algorithm. Each multiplication or addition operation is called a flop and the total number of flops represents the computational burden.

For the considered system, the computational burden of the derivative cubature Kalman filter (DCKF) is significantly reduced compared with CKF. For CKF, it is necessary to select the cubature point set to derive the nonlinear system during the time update and measurement update. However, the measurement equation of the system considered in this article is a linear equation, and choosing this method obviously increases the computational burden. The DCKF method uses KF in the linear part of the system, avoiding redundant calculations for measurement updates. Table 2 shows the detailed analysis for the computational cost of the derivative cubature Kalman filter.

Based on the above analysis, Table 2 compares the computational costs of CKF and DCKF in detail. As a result that CKF and DCKF are identical in the time update step, only the measurement update part is compared. Through the analysis of the computational cost, we can conclude that in each cycle, the CKF algorithm requires $\frac{43}{3}n^{3}+13n^{2}+4n$ flops more calculations than DCKF, while they have the comparative estimation accuracy.

**Remark** **1.** *The “flops difference” in Table 2 refers to the number of CKF flops minus the number of DCKF flops.*

### 3.4. Improvement of the Stability of the DCKF

For the DCKF algorithm, there are two main problems that may cause the filtering results to diverge. The first is that the Kalman filtering related algorithms are sensitive to some parameters. Improper parameter selection may lead to divergent estimation results. Second, the Cholesky decomposition used in the CKF algorithm requires that the decomposed error covariance matrix be a positive definite matrix. However, due to the rounding error generated by the computer during calculation, the covariance matrix may not be strictly positive definite, which will also cause the estimation result to diverge.

When the DCKF algorithm is used to calculate cubature points, it is necessary to perform Cholesky decomposition on the sample covariance matrix. Due to the randomness of the covariance matrix, ill-posed problems [41,42] are often encountered during the decomposition process, which affects the filtering performance and even causes the filtering results to diverge. Using SVD decomposition instead of Cholesky decomposition can avoid the problem of unstable filter values due to calculation errors, improve calculation efficiency, and enhance the numerical stability during calculation methods such as iterative update of the state covariance matrix. The SVD decomposition was introduced into the EKF algorithm, and a good covariance matrix iterative stability was obtained [31]. In addition, the SVD decomposition was introduced to the UKF algorithm to yield better stability [32]. The SVD decomposition is not sensitive to disturbance, and it is applied to the DCKF algorithm proposed in this paper. Compared with the CKF algorithm, the proposed SVD-FDCKF algorithm can effectively avoid the ill-posed problem of computing the square root of a covariance matrix which makes the algorithm have better stability and robustness, and obtain better filtering performance.

## 4. Simulations

Simulations are conducted in this section to comprehensively evaluate the performance of the proposed singular value decomposition-derivative CKF. The simulations are carried out in the Matlab program on a x86 Core i7 PC with 2.5-GHz CPU and 2-GB memory. The comparisons of the proposed SVD-FDCKF with the FCKF and FUKF will be made below.

### 4.1. Comparison Test of SVD-FDCKF and FCKF

In this sub-section, the state of the mobile robot system described in Section 2 is estimated using the proposed SVD-FDCKF algorithm and FCKF algorithm respectively. By comparing the simulation time and positioning results, the advantages generated by the SVD decomposition and the proposed derivative algorithm are quantitatively analyzed.

**Figure 4:**
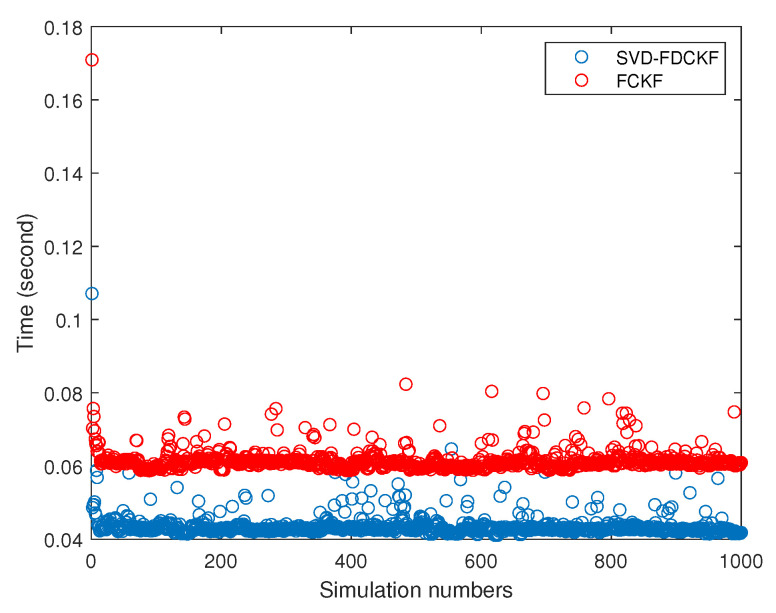
The simulation time in 1000 times Monte Carlo by the SVD-FDCKF and FCKF.

In Figure 4, the data is collected from 1000 times Monte Carlo experiments, from which we can find that the computation time of the proposed SVD-FDCKF algorithm is significantly less than that of the FCKF algorithm. The reason for the reduction in calculation time is that the SVD-FDCKF algorithm uses the characteristic that the measurement equations of the system are linear equations, avoiding complex calculations, such as the selection of cubature point sets and the propagation of cubature points. This result is consistent with the analysis of the calculation burden in Section 3, and the decrease in calculation burden leads to a reduction in calculation time. The average calculation time of the SVD-FDCKF algorithm proposed in this paper is 0.0435 s, which is 29.26% shorter than the FCKF algorithm of 0.0615 s.

**Figure 5:**
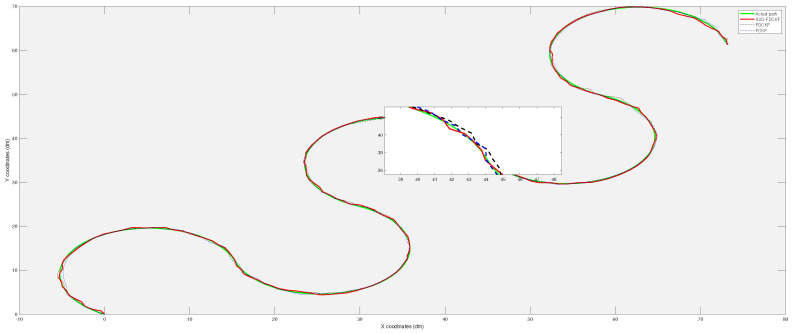
The comparison of the real trajectory of the mobile robot with the SVD-FDCKF estimated trajectory and FCKF estimated trajectory.

This set of simulations includes 200 path points. The distance *L* between the two driving wheels of the wheeled mobile robot is selected as 2 dm, which means that the width of the mobile robot is 2 dm. At the beginning of the simulation, we set the left wheel odometer change value $△M_{l,k}=1.1$, the right wheel odometer change value $△M_{r,k}=1$, and as the movement of the mobile robot, $△M_{r,k}$ becomes 1.1 and $△M_{l,k}$ becomes 1. In subsequent movements, $△M_{l,k}$ and $△M_{r,k}$ change periodically. Figure 5 shows the estimated paths of the three algorithms SVD-FDCKF, FDCKF, and FCKF. This set of simulations observes the performance of the combination of SVD decomposition and the derivative CKF by the closeness of the estimated path and the actual path. By comparing with the actual path, we can see that the three methods have similar performance in terms of positioning accuracy, which is consistent with Theorem 1 in Section 3. A part of the trajectory in Figure 5 has been randomly selected to enlarge, which is convenient to observe the path more clearly.

**Figure 6:**
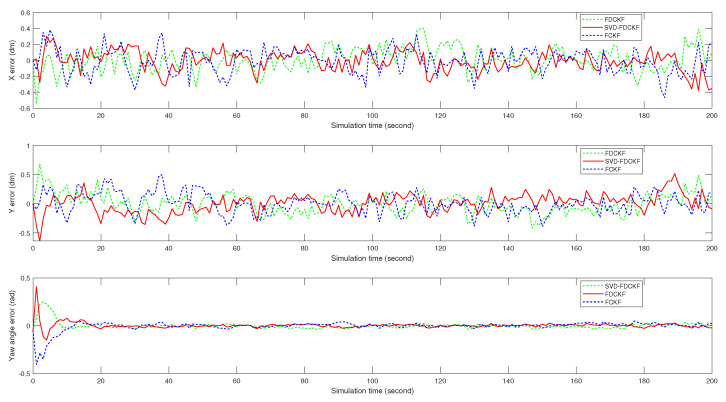
The attitude errors of mobile robots, including *x* coordinate, *y* coordinate, and yaw angle θ, are estimated by FCKF, FDCKF, and SVD-FDCKF algorithms at 200 path points, respectively.

**Figure 7:**
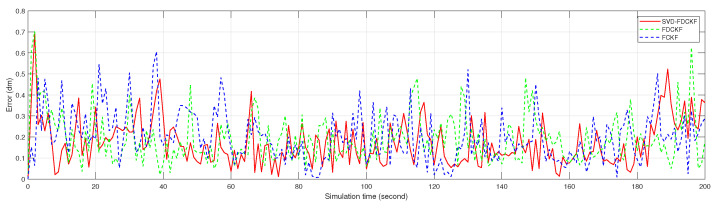
The error between the positioning result estimated by FCKF, FDCKF, SVD-FDCKF, and the actual position.

Although it is difficult to get the specific positioning error by observing Figure 5, the scale of the estimated bias seems reasonable. Therefore, we analyze the positioning errors of FCKF, FDCKF, and SVD-FDCKF in Figure 6 and Figure 7. Among them, Figure 6 shows the attitude error of the mobile robot, including *x* coordinate error, *y* coordinate error, and yaw angle error; Figure 7 shows the error of the position estimated by the three algorithms. Combining these two figures with Theorems 1 and 2, none of the algorithms have significantly higher performance than the other two algorithms. Therefore, the derivative CKF proposed in this paper reduces the computational burden of CKF without reducing the positioning error.

From Table 3, we can find that the average error and error variance of the proposed SVD-FDCKF algorithm in this paper are lower than the corresponding values of FCKF algorithm and FDCKF algorithm. It is because the process of generating cubature points requires Cholesky decomposition of the sample covariance matrix to find the square root of the matrix. If the matrix $P_{k}$ is a singular matrix, the Cholesky decomposition cannot be performed, which will cause the iterative calculation cannot continue; in addition, the Cholesky decomposition is too sensitive to the calculation error, and the small rounding error caused by the finite word length during the operation often makes the matrix $P_{k}$ loses its symmetry or positive definiteness, which results in unstable filtering results and reduces the filtering algorithm accuracy.

### 4.2. Comparison Test of SVD-FDCKF and FUKF

The unscented Kalman filter (UKF) is currently the most widely used state estimation algorithm, so it is very meaningful to compare with its performance. This sub-section uses the SVD-FDCKF and FUKF algorithms respectively for the described system to show the advantages of the proposed SVD-FDCKF algorithm through comparative experiments. The simulation results are shown in the following figures.

In this group of simulations compared with the FUKF algorithm, the UKF algorithm needs to be parameterized. This group of parameters is selected based on the previous simulation experience of filtering other models. The parameter α that determines the degree of the sigma points spread is 1; the parameter β used to describe the distribution information of the state *X* takes 1. Through these parameters, the weight $w_{i}^{\left(m\right)}$ of the sigma points and the weight $w_{i}^{\left(c\right)}$ of the covariance can be solved separately.

**Figure 8:**
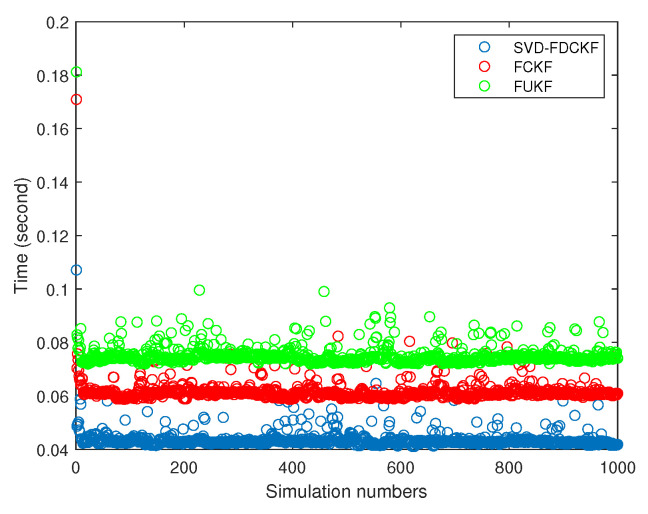
The simulation time in 1000 times Monte Carlo by the SVD-FDCKF, FCKF, and FUKF.

From the 1000 Monte Carlo experiments in Figure 8, it can be concluded that the calculation time of the SVD-FDCKF algorithm (0.0435 s) and the FCKF algorithm (0.0615 s) is lower than that of the FUKF algorithm (0.0750 s). FCKF calculation time is shorter than FUKF, it is because FUKF needs to generate 4n+2 sigma points, while FCKF only needs 4n cubature points. The reason for the short calculation time of FDCKF has been elaborated in the previous section, so it will not be repeated here.

**Figure 9:**
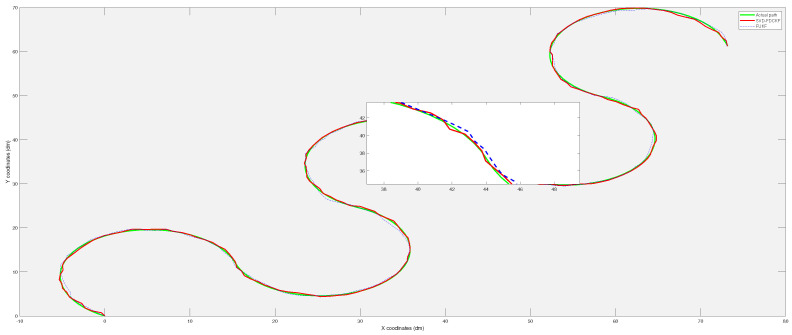
The comparison of the real trajectory of the mobile robot with the SVD-FDCKF estimated trajectory and FUKF estimated trajectory.

As shown in Figure 9, the simulation locates 200 path points. The positioning results of FUKF algorithm sometimes have large deviations, but the estimated trajectory of SVD-FDCKF algorithm is basically the consistent as the actual path. This is because the FUKF uses sigma points to approximate the probability distribution, and the cubature points used in FCKF are calculated using the third-order spherical-cubature rule, which has better stability.

**Figure 10:**
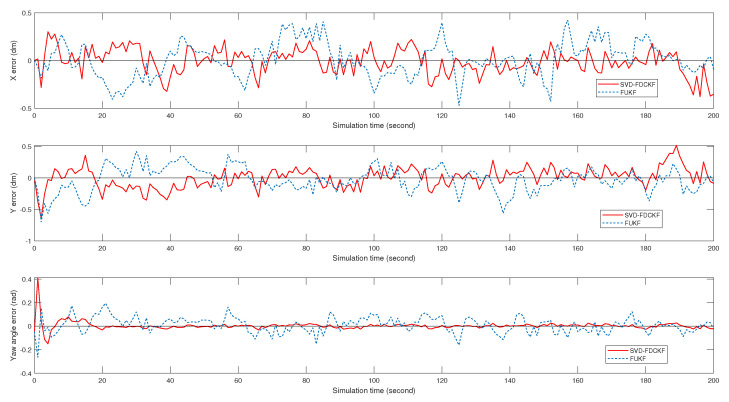
The attitude errors of mobile robots, including *x* coordinate, *y* coordinate, and yaw angle θ, are estimated by FUKF and SVD-FDCKF algorithms at 200 path points, respectively.

**Figure 11:**
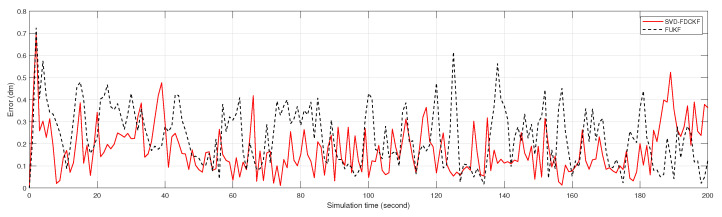
The error between the positioning result estimated by FUKF, SVD-FDCKF, and the actual position.

It can be drawn from Figure 10 and Figure 11 that for the specific mobile robot model proposed in this paper, the attitude error and overall error estimated by FUKF are significantly larger than the method proposed in this paper. Combining Table 4 and Figure 10, observe the difference of variance and the trend of attitude error. It is not difficult to find that the main reason for the larger FUKF positioning error is the poor stability of the algorithm, and the divergence of individual points affects the overall performance. The main reason for this phenomenon is that, unlike FUKF, SVD-FDCKF does not need any parameters in the state estimation process. Secondly, the weight value is positive, and finally the SVD decomposition is introduced to enhance the stability of the algorithm, thereby ensuring that the filter can run stably. Therefore, for the odometer system proposed in this paper, SVD-FDCKF has more advantages in overall accuracy and stability.

## 5. Conclusions

Based on the combination of traditional Kalman and the cubature Kalman filters under the federated filtering framework, this paper has proposed a computationally efficient Singular Value Decomposition-federated derivative cubature Kalman filtering (SVD-FDCKF) method which makes use of the ultra-wide band and inertial measurement unit information fusion to achieve high-precision indoor positioning. This algorithm can make full use of the characteristics, that the system state equation is non-linear and the observation equation is linear, to reduce the computational burden while guaranteeing the positioning accuracy. In addition, the singular value decomposition has been incorporated to the federated derivative cubature Kalman filtering method to deal with the convergence problem caused by computer rounding errors. Numerical simulations show that the proposed SVD-FDCKF method performs better than the federated cubature Kalman filter and federated unscented Kalman filter in terms of computational burden and the positioning accuracy.

The algorithm described in this article is a state estimation algorithm applied to a specific system. After a simple change of the algorithm, it can be applied to another kind of system which have linear state equation and nonlinear observation equation. The future work will be devoted to explore the robustness and stability of the proposed SVD-FDCKF. For instance, a consensus-based distributed derivative cubature Kalman filtering method will be developed to yield robust state estimation against the external disturbances.

## Figures and Tables

**Table 1:** Shorthand table of filtering algorithms.

Shorthand	Explanation
KF	A method for state estimation of linear systems.
CKF	A method for state estimation of nonlinear systems.
DCKF	Aiming at the system introduced in this article, a derivative algorithm is proposed by combining the KF and CKF algorithms. The KF algorithm is used for the linear part of the system, and the CKF algorithm is used for the nonlinear part of the system.
SVD−DCKF	The Cholesky decomposition for solving cubature points in the DCKF algorithm is replaced with SVD decomposition.
Federatedfilter	An information fusion framework.
SVD−FDCKF	Apply SVD-DCKF as a sub-filter to the federated filter.

**Table 2:** Comparison of cubature Kalman filter (CKF) and derivative cubature Kalman filter (DCKF) computational burden.

Step	CKF	DCKF	Flops Difference
Calculate the measurement cubature point	$P_{k+1|k}=S_{k+1|k}^{ckf}{S_{k+1|k}^{ckf}}^{T}$	none	$\frac{13}{3}n^{3}+n^{2}$
	$X_{k+1|k}^{*,j,ckf}=S_{k+1|k}^{ckf}\xi_{j}^{ckf}+{\hat{X}}_{k+1|k}$		
Estimate the predicted measurement	$Z_{k+1}^{j,ckf}=HX_{k+1|k}^{*,j,ckf}$	${\hat{Z}}_{k+1}=HX_{k+1|k}$	$4n^{3}-2n^{2}$
	${\hat{Z}}_{k+1}^{ckf}=\frac{1}{2n}\sum_{j=1}^{2n}Z_{k+1}^{j,ckf}$		
Calculate Kalman gain	$K_{k+1}=P_{k+1}^{XZ,ckf}{\left(P_{k+1}^{Z,ckf}\right)}^{-1}$	$K_{k+1}=\frac{P_{k+1|k}H^{T}}{HP_{k+1|k}H^{T}+R_{k+1}}$	$6n^{3}+14n^{2}+4n$
	See (26) and (27) for details		
Update states	${\hat{X}}_{k+1}^{ckf}={\hat{X}}_{k+1|k}$	${\hat{X}}_{k+1}={\hat{X}}_{k+1|k}$	×
	$+K_{k+1}^{ckf}\left(Z_{k+1}-{\hat{Z}}_{k+1}^{ckf}\right)$	$+K_{k+1}\left(Z_{k+1}-{\hat{Z}}_{k+1}\right)$	
Update error covariance	$P_{k+1}^{ckf}=P_{k+1|k}-K_{k+1}^{ckf}P_{k+1}^{Z,ckf}{K_{k+1}^{ckf}}^{T}$	$P_{k+1}=P_{k+1|k}-K_{k+1}HP_{k+1|k}$	×

**Table 3:** The average error and variance of SVD-FDCKF, FDCKF, and FCKF.

Method	Average Error/dm	Variance/dm${}^{2}$
SVD-FDCKF	0.1721	0.0119
FDCKF	0.1919	0.0138
FCKF	0.2006	0.0146

**Table 4:** The average error and variance of SVD-FDCKF and FUKF.

Method	Average Error/dm	Variance/dm${}^{2}$
SVD-FDCKF	0.1721	0.0119
FUKF	0.2344	0.0171
